# Frey’s Syndrome Surgical Treatment After Parotidectomy: A Scoping Review

**DOI:** 10.3390/jcm14020415

**Published:** 2025-01-10

**Authors:** Giorgio Barbera, Caterina Ottaviano, Guido Lobbia, Tommaso Rizzo, Esmeralda Zatta, Riccardo Nocini

**Affiliations:** 1 Head and Neck Department, Azienda Ospedaliera Universitaria Integrata di Verona, Piazzale Aristide Stefani, 1, 37126 Verona, Italy; giorgio.barbera@aovr.veneto.it (G.B.); riccardo.nocini@aovr.veneto.it (R.N.); 2 Head and Neck Department, University of Verona, Piazzale Ludovico Antonio Scuro 10, 37134 Verona, Italy; caterina.ottaviano@studenti.univr.it (C.O.); tommaso.rizzo@studenti.univr.it (T.R.); esmeralda.zatta@studenti.univr.it (E.Z.)

**Keywords:** Frey’s syndrome, surgical treatment, temporalis fascia, gustatory sweating

## Abstract

**Background/Objectives:** Frey’s syndrome surgical treatment may be either prophylactic or therapeutic. The aim of this study is to summarize the state of the art in Frey’s syndrome surgical treatment after parotidectomy and review indications, advantages, and disadvantages of different surgical options. **Materials and Methods:** The study was conducted following the PRISMA guidelines for scoping reviews; to fulfill the research enquiries, four different electronic databases (PubMed, Scopus, Cochrane, and Web of Science) were searched using the PICO protocol and key words in Frey’s syndrome surgical treatment. **Results:** A total of 15 articles met the inclusion criteria. Multiple surgical strategies have been developed over the last fifty years in an attempt to treat gustatory sweating; nevertheless, the surgical results are not always as effective as desired, and a gold standard has not been developed yet. Within the limitations of the study, a shift in surgical approaches over the years has been noted: tympanic neurectomy was the most frequently employed technique until the 1980s; more recently, local flaps interposed between the parotid tissue and skin layer have been the treatment of choice. **Conclusions**: Surgery is a viable option in Frey’s syndrome treatment, and it is indicated in cases of medical treatment inefficacy, tumor recurrence, invalidating symptoms, and unaesthetic surgical sequelae.

## 1. Introduction

Frey’s syndrome, also known as auriculotemporal syndrome or gustatory sweating, is a disorder characterized by profuse sweating, skin flushing, and warming in the preauricular and temporal area following gustatory stimulus [1]. Most of the time, Frey’s syndrome occurs after parotidectomy or submaxillary gland surgery; also, the syndrome has been observed after radical neck dissection; inflammatory, infective, and traumatic injury of the parotid region such as, for example, herpes zoster infection and condyle fractures or obstetric traumatisms with forceps [2,3]. The syndrome is due to the aberrant regrowth of facial autonomic nerve fibers: when postganglionic parasympathetic cholinergic secretomotor fibers coming from the otic ganglion are damaged by the incision of the auriculotemporal nerve, fibers previously committed to the parotid gland become misdirected, and stimulate skin glands and vessels at the time of eating and chewing [1,3].

This scoping review is specifically focused on post-parotidectomy Frey’s syndrome. To the best of our knowledge, there are no scoping reviews nor systematic ones regarding this topic. Gustatory sweating occurs in most patients undergoing parotidectomy and it usually develops in weeks or months, but it might appear up to one year after surgery [4]. Nowadays, diagnosis is performed by clinical evaluation, i.e., the presence of sweating and cutaneous flushing, and by using diagnostic tests, such as, for example, the Eisele test [5] or the Minor starch–iodine test. In the Eisele test, the patient is given a sialagogue and after several minutes, a one-ply tissue is placed on the patient’s face in order to identify the extent of the area involved in the gustatory sweating: the affected areas wet the tissue, the unaffected areas remain dry. The Minor test is the most widely applied objective test; it is considered positive when the iodine solution changes from brownish to dark purple, and negative when it does not change color.

Frey’s syndrome treatment can be either medical or surgical. While the former mainly consists of topical anticholinergics or botulinum toxin injections [6], the latter finds its rationale in interposing tissue flaps between the parotid gland and the skin to avoid anomalous fiber regeneration. Surgical treatment may be prophylactic or therapeutic [3]. Prophylactic treatment consists of adopting surgical techniques that aim at avoiding Frey’s syndrome after salivary gland surgery. In contrast, therapeutic treatment consists of executing surgical interventions after Frey’s syndrome has developed. Multiple techniques have been proposed over the last 50 years to prevent or treat gustatory sweating and the social impairment it inevitably causes. The aim of this scoping review is to present the state of the art in Frey’s syndrome surgical treatment options, to state whether a consensus among authors exists or not, and, finally, to identify any noticeable gaps in the literature. In addition, the article presents a case report of a patient suffering from Frey’s syndrome who was treated by the interposition of a temporalis muscle fascia flap.

## 2. Case Report

**Figure 1:**
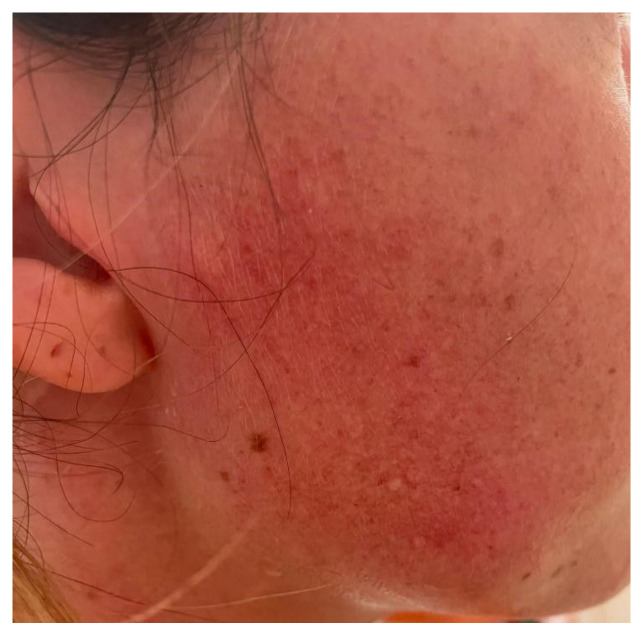
Excessive sweating and flushing syndrome after first parotidectomy.

**Figure 2:**
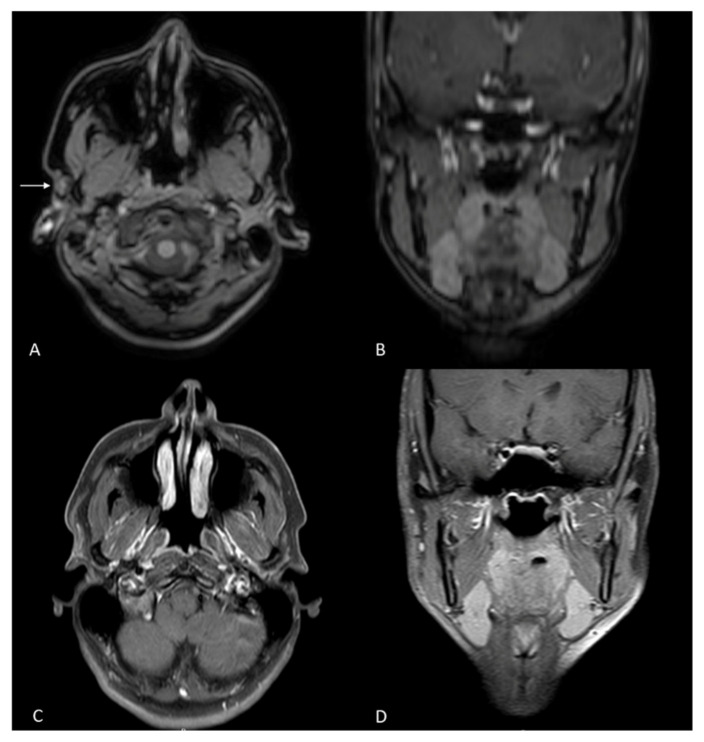
RM imaging tumor recurrence (indicated by the arrow): axial (**A**) and coronal (**B**) view. RM imaging negative for tumor recurrence at six months after secondary surgery: axial (**C**) and coronal (**D**) view.

A 20-year-old female patient presented at the outpatient section of the Maxillofacial Surgery Unit of the University of Verona. She suffered from sweating and flushing in the right preauricular area at the time of eating and chewing (Figure 1) as a surgical sequela of right superficial parotidectomy performed to remove a pleomorphic adenoma in 2015. Surgery had been performed in another hospital and the consequent Frey’s syndrome was initially treated by botulinum injection. Nevertheless, symptom resolution had not been achieved. At the time of our evaluation, the right pre-tragal region appeared swollen and suggestive of tumor recurrence. Hence, she underwent MRI, as shown in Figure 2, and echography and fine needle aspiration. These exams led to a diagnosis of pleomorphic adenoma recurrence. Treatment consisted of right superficial residual parotidectomy together with the ablation of the skin paddle overlying the tumor; the homolateral temporoparietal muscle fascia flap was raised and laid down over the surgical site. Parotidectomy was performed to remove the tumor, while a temporoparietal fascia flap was created in an attempt to resolve the gustatory sweating syndrome. Throughout the procedure, all branches of the facial nerve were preserved, the postoperative course was uneventful, and there was no functional deficit of the facial nerve.

The histological diagnosis was of a mixed salivary gland tumor. The patient underwent clinical follow-up at one, three, and six months and one year after the surgery. Six months after our surgical treatment, MRI—performed with and without contrast medium—showed no tumor recurrence. We also asked the patient to fill out a questionnaire assessing her gustatory sweating symptoms before and after the first intervention, before and after medical treatment (botulinum), and before and after secondary surgical treatment. The evaluated items were the following: the presence or absence of sweating/flushing, excessive sweating, an unpleasant smell of the sweat, and extension of the area of gustatory sweating (0.1–2.0 cm, 2.1–4.0 cm, >4.0 cm), as scored by Luna-Ortiz et al. [7]. Social impairment deriving from Frey’s syndrome was assessed, too. The results are shown in Table 1. The patient reported no significant symptom improvement after botulinum injection. For social impairment, on a scale from 1 to 10, the patient reported a score of 7/10 after the first surgery, 7/10 after the first botulinum toxin injection, and 4/10 one year after our secondary intervention, revealing a significant reduction in social discomfort. The area of gustatory sweating remained unchanged before and after botulinum injection (>4.0 cm), while it achieved an extension of less than 2.0 cm three months after secondary surgery. An unpleasant smell was never reported.

## 3. Materials and Methods

This scoping review was conducted according to the PRISMA 2020 statement guidelines for scoping reviews [8]. A summary of the protocol is provided in Table 2. A search string was created following the PICOS table, as shown in Table 2. Four electronic databases (PubMed, Scopus, Cochrane, and Web of Science) were used to search articles of interest. Inclusion criteria were the following: papers written in English without any limitation on periods of time and specifically focused on Frey’s syndrome surgical treatment; only surgical treatments performed after the parotidectomy were considered. Exclusion criteria were articles written in languages other than English, literature reviews, lack of full-text availability, articles discussing Frey’s syndrome prevention or prophylactic surgical techniques employed within parotid ablative surgery (regardless of the type of surgery, e.g., extracapsular dissection, partial superficial, total parotidectomy, etc.), Frey’s syndrome medical treatment (for example, botulinum injection and topical treatments). Inclusion and exclusion criteria were adopted to perform the first article screening; then, two of the authors (C.O. and G.L.) chose the articles on the basis of the title, abstract, and keywords. Once the final pool of articles was determined, the text of the recruited papers was read fully and summarized. Papers obtained from citation searching that were considered eligible for the study were included and were read fully and summarized, too.

## 4. Results

**Figure 3:**
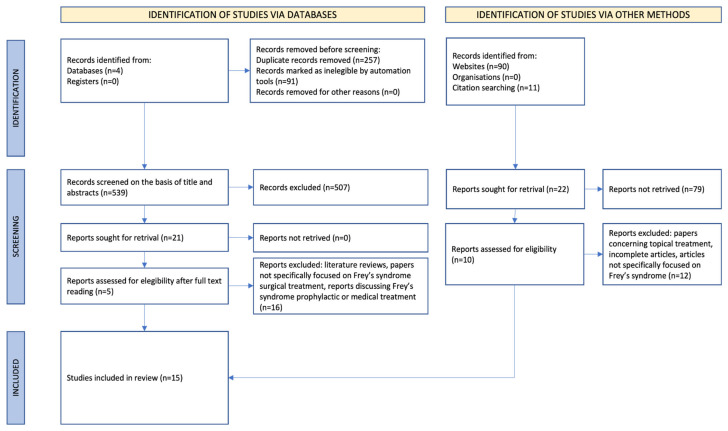
PRISMA flow diagram of paper inclusion and exclusion.

A total of 796 scientific articles written in English and without any limitations on publishing year were retrieved from four databases (Pubmed, Scopus, Cochrane, and Web of Science). After removing duplicates and after excluding records automatically identified as ineligible, the remaining 539 studies were analyzed for relevance: 507 were excluded based on keywords, title, and abstract contents and 21 were read in full by the authors in order to discuss whether to include or exclude the papers in the final pool of articles. Literature reviews, reports not specifically focused on Frey’s syndrome surgical treatment, and reports discussing Frey’s syndrome prophylactic or medical treatment were excluded (16). In the end, the final pool of articles identified by the search tools consisted of five papers. Considering 10 further articles chosen using other methods, the review was conducted on 15 papers. These articles were read fully and summarized (Table 3). The flowchart shown in Figure 3 illustrates the above-mentioned phases of the paper selection. A shift in the surgical technique prevalence was noticed over the years. While initially, tympanic neurectomy was the most employed surgical technique, more recent studies have investigated local flaps or the interposition of biomaterials between parotid tissues and the skin. Most of the papers are case reports or case series with limited sample numerosity. The indications for treatment are clear: in more than 80% of papers (13/15), the symptom severity was the reason for undergoing surgical treatment; less than 30% (4/15) advocated social impairment as one of the criteria for surgical treatment; 13% (2/15) reported parotid tumor recurrence as the rationale for performing surgery; 1 paper only considered aesthetic sequelae as a relevant indication for secondary surgical procedures. As shown in Table 3, 51% of the cases had excellent results after surgery: complete symptom relief was achieved. In 43% of the patients, gustatory symptom reduction was observed and no further treatment was required. Only 6% of the patients had an unsatisfactory outcome: their symptoms remained unchanged. Concerning the different surgical procedures, tympanic neurectomy reached an excellent result (ER) in 50%, satisfactory results (SRs) in 38%, and unsatisfactory results (URs) in 12% of cases. By using fascia lata grafts, surgeons obtained ERs in 75% and SRs in 25% of the patients. Graft interposition between the parotid gland and subcutaneous tissues allowed for ERs in 50% and SRs in 50% of the cases. All of the SRs were achieved by autologous fat injection. Temporoparietal fascia led to ERs in 56% and SRs in 44% of patients.

**Figure 4:**
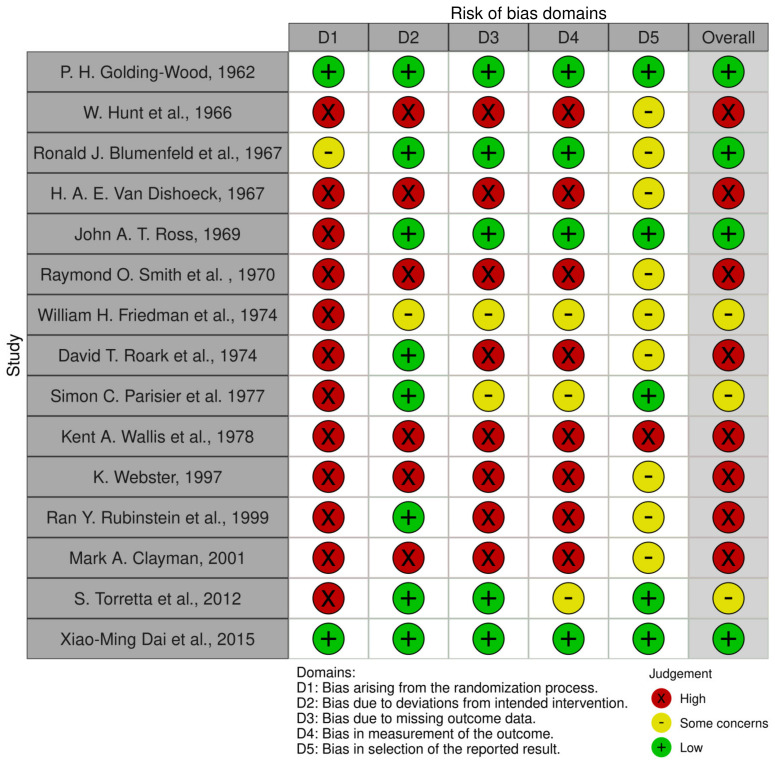
Risk of bias assessment [9,10,11,12,13,14,15,16,17,18,19,20,21,22].

**Figure 5:**
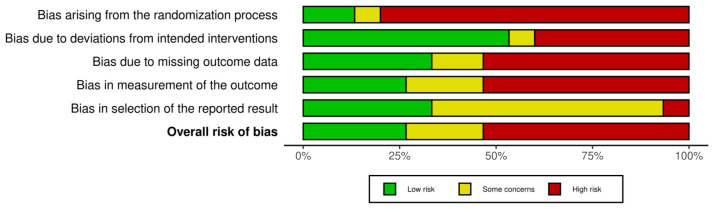
Risk of bias graph generated with robvis [23].

The risk of bias assessment (Figure 4 and Figure 5) showed an overall low or uncertain quality of the studies, particularly for case reports. There were remarkable issues with patient randomization, probably due to difficulties in identifying patients’ eligibility for Frey’s syndrome surgical treatment. Regardless, case reports by definition are prone to be biased in patient recruitment; therefore, the risk of bias in case report articles is inevitably high.

**Table 3:** Study sample and results. * Results: The table stratifies the success of the surgical approach into three grades of judgment (excellent, satisfactory, and unsatisfactory). These three judgments appear in most of the articles considered. Although a homogeneous evaluation scale does not actually exist nor has ever been validated, the authors wanted to reproduce in a more comprehensive form the results reported in the literature and create a general idea of reported success/failure rate of Frey’s syndrome surgical treatment.

Author	Year	Type of Study	Indication for Treatment	Surgical Procedure	Diagnostic Test	Patients Treated (*n*)		Results *		Duration of Follow-Up (Months)	Comments
							Excellent (*n*)	Satisfactory (*n*)	Unsatisfactory (*n*)		
P. H. Golding-Wood [24]	1962	Case report	Symptoms	Tympanic neurectomy with or without chorda tympani resection	-	3	2	1	-	18–24	Complete symptom relief in ⅔ patients, partial symptom relief in ⅓ of patients (18 months–2 years of follow up)
W. Hunt et al. [15]	1966	Case report	Social impairment	Tympanic neurectomy	-	1	-	-	1	12	Symptoms remained unchanged
Ronald J. Blumenfeld et al. [13]	1967	Retrospective Study	Social impairment	Jacobson’s Neurectomy	Minor starch-iodine test	3	2	1	-	18	All patients were relieved from their social embarrassment, a sensitive reduction of sweating area was observed (⅔ patients were free of sweating, ⅓ patients obtained a sensitive reduction of sweating)
H. A. E. Van Dishoeck [16]	1967	Case report	Symptoms severity	Tympanic neurectomy after unsuccessful tympanic plexus blockage	Minor starch-iodine test	1	1	-	-	18	Complete symptoms relief (one and a half years of follow-up)
John A. T. Ross [12]	1969	Case series	Symptoms severity	Tympanic Neurectomy	Minor starch-iodine test	5	1	4	-	3–12	Patients reported complete symptom relief immediately after surgery; progressive loss of relief was observed up to one month postoperative; unchanged results at 1 year follow-up.
Raymond O. Smith et al. [17]	1970	Case Report	Symptoms severity; social impairment	Jacobson’s Neurectomy; intratympanic division of the chorda tympani (relapse treatment)	Minor starch-iodine test	1	-	1	-	3	Symptoms improvement, no further therapy required
William H. Friedman et al. [14]	1974	Case Series	Symptoms severity	Tympanic Neurectomy	Minor starch-iodine test (only for 2 patients)	4	4	-	-	9–36	All the patients were asymptomatic with a variable follow up (minimum 9 months, maximum 3 years). Minor starch-iodine test remains positive in one case, even if he was asymptomatic.
David T. Roark et al. [18]	1976	Case Report	Symptoms severity; Recurrence following first surgery	Tympanic neurectomy, Fascia lata graft	Minor starch-iodine test	2	2	-	-	30–66	Both patients were free from symptoms and had negative minor test at the follow-up: 5.5 years and 2.5 years respectively
Simon C. Parisier et al. [11]	1977	Case series	Symptoms severity	Tympanic Neurectomy	-	6	2	2	2	8–10	33% of patients had complete symptoms resolution, 33% had symptoms improvement, 33% had no symptoms relief.
Kent A. Wallis et al. [19]	1978	Case report	Symptoms severity	Fascia lata graft	Minor starch-iodine test	2	1	1	-	9–12	Gustatory sweating relief in both of patients (50% symptom free, 50% symptom reduction), 10 and 8 months of follow up
K. Webster [20]	1997	Case Report	Gustatory sweating	Tympanic Neurectomy; Interposition of lyophilised porcine dermis sheet	One-ply facial tissue	2	1	1	-	12	1 patient had symptoms improvement by over 90% after 12 months follow up; 1 patient had no symptoms recurrence after 9 months follow up.
Ran Y. Rubinstein et al. [22]	1999	Case report	Symptoms severity; Recurrence following medical treatment	Temporoparietal fascia flap	Sweating area measurement	1	1	-	-	12	Complete relief from gustatory sweating after 1 year of follow-up. Improvement of tissue defects.
Mark A. Clayman [21]	2001	Case report	Symptoms severity; Social impairment	AlloDerm allograft, after Botox injection failure	Minor starch-iodine test	1	1	-	-	12	No symptoms, negative Minor’s test (1 year follow-up)
S. Torretta et al. [10]	2012	Case Series	Symptoms severity	Autologous Fat Injection (AFI)	Minor starch-iodine test; Luna-Ortiz score	4	-	4	-	3–12	Average reduction of gustatory sweating area: −85.3%. Average Luna Ortiz score variation: −74%. Procedure is minimally invasive and might be performed under local anesthesia.
Xiao-Ming Dai et al. [9]	2015	Retrospective cohort	Symptoms severity and unesthetic facial asymmetry	Interposition sternocleidomastoid flap overlapped with temporalis fascia flap/graft	Minor starch-iodine test	17	9	8	-	6–52	After treatment, average reduction of gustatory sweating area was 90%; skin erythema disappeared in all patients
**Total**						**53**	**27 (51%)**	**23 (43%)**	**3 (6%)**	**-**	

## 5. Discussion

Frey’s syndrome surgical treatment is a rather controversial topic for a multiplicity of reasons. There are no randomized trials on the issue, but only case reports, case series, and few literature reviews; moreover, most of the scientific articles available through search tools are mainly concerned on gustatory sweating medical treatment rather than surgical approaches.

Frey’s syndrome diagnosis is performed with an objective test, the Minor test. In their literature review, Clayman et al. reported that the Minor test identified Frey’s syndrome in over 90% of patients who underwent a parotidectomy, even if they were asymptomatic [3]. Regardless, the frequency varies significantly in the literature: Luna-Ortiz et al. reported a frequency of Frey’s syndrome after parotidectomy varying from 5% to 100% and estimated a 66% worldwide incidence [7]. According to Mantelakis et al., Frey’s syndrome is present in almost 100% of patients subjected to parotidectomy, even though only 10 to 30% of patients independently report and seek help for symptoms, while 20 to 60% of them admit gustatory sweating presence when asked [4]. Similarly, De Bree et al. reported 10% of patients who spontaneously complain about Frey’s syndrome symptoms and 30% to 40% of patients admitting gustatory sweating when interviewed [25]. Hence, Frey syndrome has a relatively subjective nature that should be carefully considered by surgeons at the time of determining whether to undergo surgical treatment or not. In this sense, Luna-Ortiz et al. have proposed an evaluation scale that takes into consideration cervicofacial extension of the hyperhidrotic area (assessed by the Minor test), intensity in terms of the perception of sweating and the presence of an unpleasant sweat smell [7]. We administered our patient a questionnaire assessing the Frey’s syndrome symptom severity following the Luna-Ortiz scale. It is likely to affirm that this evaluation scale should be considered a useful system to better identify patients’ eligibility for treatment and to choose the most appropriate therapeutic method.

Indeed, indications for surgical treatment still need to be clearly defined, and the present study highlighted the main reasons for undergoing surgical treatment of gustatory sweating. Undoubtedly, surgery should be considered in severely symptomatic patients no longer responding to medical therapy [4]. In these cases, surgery is adopted with the purpose of avoiding further secondary procedures. Secondly, patients affected by gustatory sweating and diagnosed with parotid tumor recurrence are eligible for surgery to perform both tumor resection and Frey’s syndrome treatment in a one-stage surgical procedure [9]. This is the scenario of our clinical case: the patient had both Frey’s syndrome presence and tumor recurrence. Furthermore, surgical treatment might be indicated to address auriculotemporal syndrome in the presence of facial and upper cervical contour defects or depressions deriving from previously performed parotidectomy. Both functional and aesthetic issues are simultaneously solved by a surgical approach [9,10].

Multiple surgical techniques have been developed to address gustatory sweating. The current study has revealed a shift in surgical technique prevalence over the years. While, until the 1980s, tympanic neurectomy was the most employed surgical technique, more recent studies have investigated local flaps or the interposition of biomaterials between parotid tissues and the skin.

Tympanic neurectomy is reported as the treatment of choice in over 50% of the studies included in this scoping review. In their case series on a sample of six patients, Parisier et al. reported that tympanic neurectomy achieved complete symptom resolution in one-third of patients, satisfying results in one-third, and no symptom relief in the remaining one-third [11]. In his case series on five patients, Ross reported the immediate relief of symptoms in all cases and a progressive partial recurrence of symptoms within the first month postoperation, remaining stable one year after surgery [12]. Friedman et al. as well as Blumfeld et al. reported successful results in the long term after tympanic neurectomy [13,14]. According to the literature review by Hays et al. (73 case reports), tympanic neurectomy achieved complete symptom resolution in 56% of patients, satisfactory results not requiring further treatment in 26% of patients, and unsatisfactory results that needed secondary surgery in 18% [26]. The tympanic neurectomy technique consists of the resection of the tympanic nerve through trans-meatal incision after entering the middle ear. Tympanic neurectomy may be performed alone or combined with or followed by chordae tympani resection in cases of symptom recurrence [11,15,16,26]. Bone chips or bone wax have been employed by some authors to obliterate the bed of the tympanic plexus in order to avoid nerve regeneration [13,26]. Neurectomies have been gradually abandoned as there are considerable drawbacks such as developing gustatory sweating recurrence, developing xerostomia, and losing gustatory taste [13,17].

Fascia lata graft is another surgical approach to address Frey’s syndrome. It consists of positioning a fascia lata graft underneath a cutaneous–subcutaneous facial flap, raised in the gustatory sweating area. The donor site is the patient’s thigh. Even though limited evidence of success is reported among the authors (four case reports only), fascia lata graft seems to be a valid barrier to avoid anomalous nerve regeneration; fascia lata comes from the autologous tissues of the lower limbs, it is easy to harvest, thicker than other similar anatomic structures, and the donor site rarely causes disabilities in movements. Despite two surgical sites being required, increasing the risk of complications, high satisfaction rates have been documented in the literature [18,19,27]. All the patients treated by Roark et al. and Wallis et al. registered complete symptom relief after fascia lata graft surgery; therefore, the authors considered it an excellent remedy to Frey’s syndrome. Roak et al. concluded their article on fascia lata graft/flap, concluding that, considering their limited experience in the use of subcutaneous fascia lata grafts, they are not capable of determining the superiority of this surgical approach in comparison to tympanic neurectomy and chordae tympani neurectomy [18]. Regardless, the authors consider fascia lata graft a reliable, safe technique, the efficacy of which should be further tested; the fascia lata flap will eventually acquire more efficacy in gustatory sweating treatment [18].

Allografts are a viable option for treating anomalous re-innervation. Allografts may be obtained from cadaver skin—deprived of epidermal and dermal cells—or lyophilized porcine dermis. The advantages of this technique are the following: limited dissection of the operative site and absence of a donor site [20,21]. Porcine dermis causes no immunological response, no rejection, and minimal inflammation of the tissues; hence, it might be considered a low-complication-rate technique [20,21]. Encouraging results were registered by Clayman and Webster: symptoms disappeared in almost all of their treated patients.

Autologous adipose fat injection (AFI) is a minimally invasive procedure used in parotidectomy sequelae for aesthetic and functional purposes. Fat creates a barrier between the parotid bed and the cheek layer. AFI can be performed in a one-day surgery setting and under local anesthesia and sedation. It must be preceded by carefully marking the sweating area. The rationale of this technique is to gently distribute adipocytes into the immediate subdermal plane, following different directions, and creating a barrier to prevent abnormal nerve neo-anastomosis to the sweat glands [28]. Despite the numerous advantages, AFI may need to be repeated to achieve satisfying results. Torretta et al. reported four cases treated by adipocyte fat injection, resulting in a 85,3% reduction in the gustatory sweating area and a 74% reduction in the Luna-Ortiz score. Frey’s syndrome symptoms went from severe to mild [10].

In our experience, we adopted a temporoparietal fascia flap to treat our patient, obtaining a reduction in the sweating and flushing cutaneous area and improvement in social distress, simultaneously. The facial nerve was preserved, and no drawbacks followed the surgical treatment. A single surgical site was utilized. The symptom severity, failure of medical treatment, social impairment, and parotid recurrence were the reasons that guided our choice towards the surgical approach. Temporoparietal fascia together with sternocleidomastoid muscle are examples of local barriers interposed between the cheek skin and parotid gland tissues. A temporoparietal fascia flap is a local pedicled flap indicated in cases of medical treatment failure. Rubinstein et al. reported a case of gustatory sweating resolution and simultaneous cheek contour improvement. This technique is highly beneficial for the patient; it is based on a local flap close to the parotid gland bed and a second surgical site is not required. The drawbacks related to this technique are the risk of injury to the frontal branch of the facial nerve and transient or permanent alopecia in the temporal region [22]. Temporalis fascia was adopted by Dai et al. in a sample of 17 patients. Temporoparietal fascia was applied as a flap, or a graft overlapped with a sternocleidomastoid muscle flap to create a double layer, obtaining a 90% symptom reduction and significant reduction in the area of gustatory sweating. The double layer provides a thicker barrier against abnormal reinnervation and better aesthetic correction of mandibular contour; furthermore, local flap avoids a secondary surgical site and is free from rejection risk. Finally, the temporalis fascia flap is less prone to postoperative infections in comparison to the graft, due to the presence of a vascular pedicle [9]. The risk of facial nerve injury can be avoided by identifying the course of the frontal nerve branch and by carefully limiting the anterior extent of the temporoparietal fascia flap elevation [22].

A lack of consensus in Frey’s syndrome surgical treatment emerges from the present study. This is probably due to the limited number of studies and limited evidence-based efficacy of surgical approaches that have been demonstrated to have a level of evidence between 4 and 5 [4]. According to current literature, Frey’s syndrome medical management is considered the first-line therapy in gustatory sweating treatment. Medical treatment is safe and effective, though sometimes insufficient in achieving symptom regression. Notwithstanding, the role of surgery in Frey’s syndrome treatment has not been clearly established yet. Surgery is unfrequently suggested: it is reserved for cases in which conservative or medical treatment have failed or insufficient symptom improvement has been achieved [29]. Finally, surgical management has an increased risk of facial nerve injury. Nevertheless, today, all of the above-mentioned techniques seem to guarantee partial to complete symptom resolution, together with significant aesthetic improvement. The results extrapolated from this literature review reveal highly satisfactory outcomes in Frey’s syndrome surgical treatment: through surgical treatment, 94% of patients reached gustatory symptom reduction or resolution.

## 6. Conclusions

The findings of the present study permit the following conclusions to be drawn. Frey’s syndrome surgical management is indicated in the case of medical treatment failure and may be highly beneficial in cases of disease recurrence and unsolved social impairment deriving from both sweating and unaesthetic parotidectomy sequelae.

Considering the limitations of the present study, which are due to the lack of randomized trials on the topic and the prevalence of case reports and case series over literature reviews, further studies with a larger sample numerosity and standardized surgical protocols are firmly recommended to enhance the evidence of efficacy in Frey’s syndrome surgical management.

## Figures and Tables

**Table 1:** Frey’s syndrome assessment questionnaire results.

	After Primary Surgery	After Botulinum Toxin Injection	Immediately After Secondary Surgery	3 Months After Secondary Surgery	6 Months After Secondary Surgery	1 Year After Secondary Surgery
Clinical manifestation	Yes	Yes	Yes	Yes	Yes	Yes
Extent of the affected area 0.1–2.0 cm 2.1–4.0 cm >4.0 cm	>4.0 cm	>4.0 cm	0.1–2.0 cm	0.1–2.0 cm	0.1–2.0 cm	0.1–2.0 cm
Excessive focal sweating	Yes	Yes	Yes	Yes	Yes	Yes
Unpleasant smell sweating	No	No	No	No	No	No
Social impairment (0/10)	7/10	7/10	5/10	4/10	4/10	4/10

**Table 2:** (A) Summary of the study protocol. (B) Keywords for the search string according to the PICO protocol.

A		
1	Protocol and registration	Not available
2	Eligibility criteria	Peer-reviewed journal papers; publication period: up to 2024; language: English; topic: Frey’s syndrome surgical treatment following parotidectomy.
3	Information sources	PubMed, Scopus, Cochrane, Web of Science
4	Search	PICOS search strategy.
5	Source of evidence and data charting	All the literature retrieved was screened by the authors. Papers discussing surgical treatment of Frey’s syndrome were included in the scoping review, whereas papers focusing on medical or alternative treatments were not taken into consideration.
6	Data items	Authors, year of publication, article format, indication for surgery, surgical technique employed, diagnostic test, results.
7	Synthesis of results	Information about indications, surgical technique, and result assessment in Frey’s syndrome surgical treatment were extrapolated from the articles and synthesized. Assessment of long-term results was attributed a score ranging from excellent to satisfactory to unsatisfactory considering that most of the articles expressed treatment success in such terms.
**B**		
Population		Text word (1): “Frey’s syndrome” OR “Frey Syndrome” Mesh Terms (2): “Sweating” OR “Gustatory”
Intervention		Text words (3): “surgical treatment”
Comparison		Text words (4): “botulinum” Text words (5): “medical treatment”
Outcomes		Text words (6): “long-term results”
Study design		MeSH term (7): “Systematic review”
Search string		(1) AND (2) AND (3) NOT (4) NOT (5) AND (6) AND (7)

## Data Availability

This is a review of literature; no new data were created in the present work.

## References

[1] Li C., Wu F., Zhang Q., Gao Q., Shi Z., Li L. (2015). Interventions for the Treatment of Frey’s Syndrome. Cochrane Database Syst. Rev..

[2] Anesi A., Di Bartolomeo M., Pellacani A., Ferretti M., Cavani F., Salvatori R., Nocini R., Palumbo C., Chiarini L. (2020). Bone Healing Evaluation Following Different Osteotomic Techniques in Animal Models: A Suitable Method for Clinical Insights. Appl. Sci..

[3] Clayman M.A., Clayman S.M., Seagle M.B. (2006). A Review of the Surgical and Medical Treatment of Frey Syndrome. Ann. Plast. Surg..

[4] Mantelakis A., Lafford G., Lee C.W., Spencer H., Deval J.-L., Joshi A. (2021). Frey’s Syndrome: A Review of Aetiology and Treatment. Cureus.

[5] Eisele D.W. (1992). Simple Method for the Assessment of Frey’s Syndrome. Laryngoscope.

[6] Bertossi D., Giampaoli G., Lucchese A., Manuelli M., Albanese M., Nocini R., Nocini P.F. (2019). The Skin Rejuvenation Associated Treatment—Fraxel Laser, Microbotox, and Low G Prime Hyaluronic Acid: Preliminary Results. Lasers Med. Sci..

[7] Luna-Ortiz K., Sansón-RíoFrío J.A., Mosqueda-Taylor A. (2004). Frey Syndrome. A Proposal for Evaluating Severity. Oral Oncol..

[8] Page M.J., McKenzie J.E., Bossuyt P.M., Boutron I., Hoffmann T.C., Mulrow C.D., Shamseer L., Tetzlaff J.M., Akl E.A., Brennan S.E. (2021). The PRISMA 2020 Statement: An Updated Guideline for Reporting Systematic Reviews. BMJ.

[9] Dai X.-M., Liu H., He J., Tu M.-S., Yu L.-F., Liu L. (2015). Treatment of Postparotidectomy Frey Syndrome with the Interposition of Temporalis Fascia and Sternocleidomastoid Flaps. Oral Surg. Oral Med. Oral Pathol. Oral Radiol..

[10] Torretta S., Pignataro L., Capaccio P., Brevi A., Mazzola R. (2012). Fat Injections for the Management of Post-Parotidectomy Frey Syndrome: A Technical Note. J. Craniomaxillofac. Surg..

[11] Parisier S.C., Blitzer A., Binder W.J., Friedman W.F., Marovitz W.F. (1978). Evaluation of Tympanic Neurectomy and Chorda Tympanectomy Surgery. Otolaryngology.

[12] Ross J.A. (1970). The Function of the Tympanic Plexus as Related to Frey’s Syndrome. Laryngoscope.

[13] Blumenfeld R.J., Friedman J.E. (1967). Intratympanic Surgical Treatment of Frey’s Syndrome. Arch. Otolaryngol..

[14] Friedman W.H., Swerdlow R.S., Pomarico J.M. (1974). Tympanic Neurectomy: A Review and an Additional Indication for This Procedure. Laryngoscope.

[15] Hunt W., Joseph D., Newell R., Hanna H.H. (1966). Gustatory Sweating. Report of a Case Treated by Tympanic Neurectomy. Arch. Otolaryngol..

[16] Van Dishoeck H.A. (1968). The Auriculo-Temporal or Frey Syndrome and Tympanic Neurectomy. Laryngoscope.

[17] Smith R.O., Hemenway W.G., Stevens K.M., Ratzer E.R. (1970). Jacobson’s Neurectomy for Frey’s Syndrome. Am. J. Surg..

[18] Roark D.T., Sessions R.B., Alford B.R. (1975). Frey’s Syndrome-a Technical Remedy. Ann. Otol. Rhinol. Laryngol..

[19] Wallis K.A., Gibson T. (1978). Gustatory Sweating Following Parotidectomy: Correction by a Fascia Lata Graft. Br. J. Plast. Surg..

[20] Webster K. (1997). Early Results Using a Porcine Dermal Collagen Implant as an Interpositional Barrier to Prevent Recurrent Frey’s Syndrome. Br. J. Oral. Maxillofac. Surg..

[21] Clayman M.A., Clayman L.Z. (2001). Use of AlloDerm as a Barrier to Treat Chronic Frey’s Syndrome. Otolaryngol. Head Neck Surg..

[22] Rubinstein R.Y., Rosen A., Leeman D. (1999). Frey Syndrome: Treatment with Temporoparietal Fascia Flap Interposition. Arch. Otolaryngol. Head Neck Surg..

[23] McGuinness L.A., Higgins J.P.T. (2021). Risk-of-bias VISualization (Robvis): An R Package and Shiny Web App for Visualizing Risk-of-bias Assessments. Res. Synth. Methods.

[24] Golding-Wood P.H. (1962). Tympanic neurectomy. J. Laryngol. Otol..

[25] De Bree R., van der Waal I., Leemans C.R. (2007). Management of Frey Syndrome. Head Neck.

[26] Hays L.L. (1978). The Frey Syndrome: A Review and Double Blind Evaluation of the Topical Use of a New Anticholinergic Agent. Laryngoscope.

[27] Giovannetti F., Barbera G., Priore P., Pucci R., Della Monaca M., Valentini V. (2019). Fascia Lata Harvesting: The Donor Site Closure Morbidity. J. Craniofac. Surg..

[28] Mazzola R.F., Cantarella G., Torretta S., Sbarbati A., Lazzari L., Pignataro L. (2011). Autologous Fat Injection to Face and Neck: From Soft Tissue Augmentation to Regenerative Medicine. Acta Otorhinolaryngol. Ital..

[29] Young A., Okuyemi O.T. (2024). Frey Syndrome. StatPearls.

